# Patients’ and clinicians’ views on the appropriate use of safety-netting advice in consultations—an interview study from Sweden

**DOI:** 10.1136/bmjopen-2023-077938

**Published:** 2023-10-05

**Authors:** Rita Fernholm, Carolina Wannheden, Sofia Trygg Lycke, Sara Riggare, Karin Pukk Harenstam

**Affiliations:** 1 Department of Neurobiology, Care Sciences and Society, Karolinska Institutet, Stockholm, Sweden; 2 Department of Learning, Informatics, Management and Ethics, Medical Management Centre, Karolinska Institutet, Stockholm, Sweden; 3 Department of Women's and Children's Health, Participatory eHealth and Health Data, Uppsala University, Uppsala, Sweden; 4 Department of Women's and Children's Health, Karolinska Universitetssjukhuset, Stockholm, Sweden

**Keywords:** Primary Health Care, Clinical Decision-Making, Clinical Reasoning, Risk management, Patient-Centered Care

## Abstract

**Abstract:**

**Background:**

A promising approach to manage clinical uncertainty and thereby reduce the risk of preventable diagnostic harm is to use safety-netting advice (ie, communicating structured information to patients about when and where to reconsult healthcare).

**Aim:**

To explore clinicians’ and patients’ views on when and how safety-netting can be successfully applied in primary-care and emergency-care settings.

**Design and setting:**

An exploratory qualitative research design; we performed focus groups and interviews in a Swedish setting.

**Participants:**

Nine physicians working in primary or emergency care and eight patients or caregivers participated. The participants were an ethnically homogeneous group, originating from Western European or Australian backgrounds.

**Method:**

Data were analysed inductively, using the framework method. The results are reported according to the Standards for Reporting Qualitative Research guidelines for reporting qualitative research.

**Results:**

In order to manage diagnostic uncertainty using safety-netting, clinicians and patients emphasised the need to understand the *preconditions* for the consultation (ie, the healthcare setting, the patient’s capacity and existing power imbalance). Furthermore, participants raised the importance of *establishing a mutual understanding* regarding the patient’s perspective and the severity of the situation before engaging in safety-netting advice.

**Conclusion:**

The establishment of a shared mental model between clinician and patient of the preconditions for the clinical encounter is a vital factor affecting how safety-netting advice is communicated and received and its ability to support patients in problem detection and planning after the visit. We suggest that successful safety-netting can be viewed as a team activity, where the clinician and patient collaborate in monitoring how the patient’s condition progresses after the care visit. Furthermore, our findings suggest that to be successfully implemented, safety-netting advice needs to be tailored to the clinical context in general and to the patient–clinician encounter in particular.

**Strengths and limitations of this study**

- The study includes views from both patients and clinicians into the experiences of how clinical uncertainties can be managed with safety-netting advice.
- The study adds to mid-range theory of patient safety management by exploring safety-netting as a shared cognitive task between patients and clinicians.
- The results reflect the experiences of the participants and therefore other aspects of safety-netting might exist that were not discussed during the interviews.

## Introduction

 In primary healthcare (PHC) and the emergency department (ED), patients often consult for new symptoms and the diagnosis is not known. In these contexts, there is a high concentration of decision-making regarding diagnoses, and more diagnostic errors are reported compared with other parts of the healthcare system with lower contact volume (ie, lower exposures to undiagnosed patients). In Sweden, the reports of patient harm from these settings often involve diagnostic errors, for example missed fractures or cancer.^1^ Preventable harm due to diagnostic error often have more severe consequences than harm due to other causes,^2^ and the problem is global.^3^ Few interventions to decrease the risk of diagnostic errors have been tested,^4^ and to the best of our knowledge, none have been implemented systematically. A promising intervention is structured safety-netting advice as a tool for managing clinical uncertainty, with the aim of protecting patients from harm due to diagnostic error by empowering appropriate reconsultations and thereby still receive adequate treatment in due time. Safety-netting advice includes the communication of four central pieces of information: (a) the existence of some uncertainty regarding the diagnosis, (b) what to expect about the timeline of events, (c) what symptoms to look out for and (d) when/how to seek further help.^5^ Although the implementation of safety-netting advice has the potential to improve patient safety through appropriate reconsultation, there is a mismatch between best practice of the use of safety-netting and clinical practice in part due to lack of time and knowledge.^6 7^ Certain qualitative studies concerning acutely ill children reveal that safety-netting is instinctively employed in PHC, highlighting the necessity for a comprehensive and customised approach to safety-netting.^8 9^ There is a general lack of studies investigating patients’ experiences of how clinicians communicate diagnostic uncertainty and use of safety-netting advice in clinical encounters. Some work has been done, indicating that patients and clinicians had a shared understanding of issues considered important, like communicating openly and ensure the possibility to reconsult,^10^ and that patients can be willing to accept responsibility with clear guidance.^11^

The aim of this study was to explore clinicians’ and patients’ views on when and how safety-netting can be applied successfully in primary- and emergency-care settings.

## Method

### Study setting

The study is based on an integration of the perspectives of patients, informal caregivers and clinicians working in primary and emergency care from different regions in Sweden.

### Study design

An exploratory research design was used, based on focus-group discussions and individual interviews with clinicians, patients and informal caregivers. By combining these two techniques, we were able to capture both group reflections and individual insights. This enabled patients to talk freely when there were no doctors present and vice versa. The research group comprised health services researchers with expertise in qualitative research methodology. Two of the researchers were clinicians (one general practitioner (GP) and one paediatrician) and two were health informaticians, where one was also a patient researcher with expertise in participatory medicine. The Standards for Reporting Qualitative Research guidelines were used for reporting.

### Study participants

We included 17 participants: eight patients and informal caregivers (where five female) with experience of uncertain diagnoses and serious diseases, and nine physicians (where seven female) with experience of working with diagnostic uncertainties in primary care or in emergency care (table 1). The group exhibited ethnical homogeneity, originating from Western Europe and Australia. The study participants were purposefully recruited to capture a variety of stakeholder perspectives.^12^ Patients and informal caregivers were recruited through a patient network and a cancer patient organisation, both with experience of collaborative improvement work in healthcare. Clinicians were recruited from the Swedish Association of General Practice and Swedish Society for Paediatric Emergency Medicine. We aimed for variety in care settings (primary care, out-of-hours services and emergency care), experience (residents and specialists, minimum 4 years of experience) and specialities (adult and paediatric care). All participants signed an informed consent to participate and had the right to withdraw at any time without stating a reason. Patients and informal caregivers were reimbursed to compensate for loss of income, in line with recommendations provided by the Swedish Association of Local Authorities and Regions. Based on the concept of information power,^13^ we determined our sample size sufficient to answer our research question.

**Table 1:** Participant characteristics (with a range of 5–35 years of experience as physician/patient/informal caregiver)

ID	Role/organisation	Gender female/male	Age
L01	Physician, primary care	f	39
L02	Physician, primary care	f	46
L03	Physician, primary care	f	54
L04	Physician, emergency care	f	47
L05	Physician, primary care and emergency care	f	43
L06	Physician, primary care	f	42
L07	Physician, primary care	m	52
L08	Physician, primary care	m	35
L09	Physician, primary care	f	61
P01	Patient	f	61
P02	Patient	f	57
P03	Informal caregiver	m	70
P04	Patient	f	55
P05	Patient	m	47
P06	Patient	f	46
P07	Patient	m	48
P08	Patient	f	52

### Data collection

The data collection period lasted from November 2020 to April 2021.

All participants were invited to participate in an initial focus group discussion, followed by an individual interview (onlinesupplemental files 1^3^): 17 agreed to participate in a focus group or interview, 14 participated in the focus group only and seven participated in an interview only. None of the patients had met any of the clinicians before. Initially, we performed two heterogeneous focus group discussions (seven participants per group, involving both clinicians and patients/informal caregivers). Both sessions started with showing a short video clip (5 min) in which one of the authors (RF) introduced the safety-netting concept, followed by a compilation of clinician and patient voices describing experiences of diagnostic uncertainty. Based on this video clip, the participants were invited to reflect on how diagnostic thinking and safety-netting behaviours among patients and clinicians could be supported. The discussion was facilitated by RF based on a topic guide with probing questions and lasted for 90 min in each session. Three authors (KPH, CW and SR), participated as observers and took notes.

After a preliminary analysis of these focus group discussions, guides were developed for semistructured interviews with clinicians and patients, inspired by the approach of cognitive task analysis.^14^ The purpose of these interviews was to explore their experiences and views on safety-netting in more depth and give the opportunity to speak freely, without anyone from the other category of participants present. After watching the same video clip that was shown in the focus group discussions, the participants were invited to reflect on a specific incident they had experienced where safety-netting would have been appropriate due to diagnostic uncertainty. The interviews lasted approximately 45 min on average and were performed by RF, KPH and STL. During the last interviews, no new information emerged. Due to the COVID-19 pandemic, data were collected and recorded via Zoom’s video conferencing platform.

### Patient and public involvement

The development of the research question was informed by patients’ priorities, experiences and preferences by earlier work by the researchers^15^ and by the participating of a patient (SR) among the authors. SR has extensive experience as a patient and was involved in the study design and the recruitment of participating patients, as well as the rest of the study. The results will be disseminated to study participants by e-mail.

### Data analysis

The interviews were transcribed verbatim and pseudonymised with coded labels indicating the type of respondent (physician, patient or informal caregiver). Authors RF, KPH, STL and CW collaboratively conducted an inductive qualitative content analysis, using procedures suggested by Graneheim and Lundman.^16 17^ First, they familiarised themselves with the transcripts individually by reading them in detail and identifying meaning units, which they compared and discussed in meetings, one transcript at a time. The authors then collaboratively condensed these meaning units and labelled them with codes. CW organised the data into the following columns in a spreadsheet: transcript ID, role (patient, physician and informal caregiver), meaning unit, condensed meaning unit and code. Thereafter, CW transferred the rows from the spreadsheet into a mind-mapping software package (FreeMind V.1.0.1), which facilitated the abstraction process involving the iterative relabelling and grouping of codes into categories and subcategories. The authors had several meetings to interpret the data until agreement was reached on a sufficient level of abstraction. Reflexivity was integrated in the analytic process through iterative occasions where the possible impact of subjective experiences and knowledge of the researchers on the interpretations of the findings where discussed. To illustrate our findings, representative quotes were translated from Swedish into English.

## Results

The analysis resulted in two main categories: (1) understanding and acknowledging the preconditions for the consultation and (2) Establishing a mutual understanding.

### Category 1: understanding the preconditions for the consultation

When reflecting on the concept of safety-netting, the participants raised the importance of first understanding the preconditions for the consultation, which includes the healthcare setting, the individual patient’s capabilities and the imbalance of power in the consultation. The preconditions relate to all steps of the consultation: before, during and after the actual meeting.

#### The healthcare setting

Clinicians explained that the choice of safety-netting advice depended on the level of care, due to variations in both the issues the patient wanted addressed and the availability of resources. Primary care clinicians seemed to prefer to schedule physical or telephone consultations for follow-up, rather than providing the patient with safety-netting advice. Continuity of care (follow-up by the same doctor) was central to them and provided a way to build safety-netting into the care processes, rather than shifting responsibility to the patient. In contrast, emergency-care clinicians emphasised the importance of giving safety-netting advice because they had no opportunity to schedule follow-up visits. However, due to the difficulty of providing sound advice, some explained that they sometimes encouraged patients to reconsult if their condition deteriorated, rather than giving detailed safety-netting advice.

The clinicians agreed that the type of safety-netting given strongly depend on access to care. If no follow-up care was scheduled, patients needed to be able to make contact with care providers quickly to reconsult if their condition did not develop as expected. The patients’ reflection on different healthcare settings was that it can be difficult to know which is the appropriate level of care, and that sometimes they need to go to the ED due to lack of availability at their primary care centre.

And I’m also thinking that safety-netting advice is very much dependent on where you are. […] If I’m at the out-of-hours office it’s pretty simple, concrete advice. (Focus group, physician)

#### The patient’s capabilities

Clinicians agreed that it is important to support patients in taking responsibility for their own care trajectory and emphasised that patients are not all the same. Different patients are able (and willing) to take responsibility on different levels—depending on their knowledge, experience and condition. For example, it was suggested that it may be challenging to shift safety-netting responsibility to older adults with multimorbidity and polypharmacy, and in these cases, clinicians preferred to book a follow-up visit or, if in doubt, admit the patient for observation. To increase their capacity, patients emphasised the value of involving their social network, including informal caregivers and patient organisations, who could help them make sense of the information they received from healthcare providers.

Then I also think, if you can engage the patient in any way in this situation, you know, people who are sick are not only their illness. Instead, they’re people with an illness and have abilities…which differ from one person to another. But if you find a way to engage them so the patient can help in any way, then I think that’s beneficial too. (Focus group, physician)

#### Imbalance of power

There was a discussion about how interactions are influenced by asymmetry in the patient–clinician relationship, including the imbalance of power (ie, the patient has a dependency relationship to the clinician), language asymmetry (ie, the use of medical jargon can make it difficult for patients to understand) and information asymmetry (ie, clinicians and patients have access to different information). Given their vulnerable position, patients expressed a need to be seen and heard by their clinicians. Patients underscored the significance of doctors actively listening, treating patients with respect, and acknowledging their concerns and emotions.

[…] like what was described in the film, the superiority and the inferiority. That… that the person who is inferior needs encouragement to ask the doctor questions. And also, I think, it’s important to see the human being. That’s why I think it’s so important that the patient sees the doctor as a person. And that the doctor sees the patient as a person. That’s just a pure humanistic opinion I have. (Focus group, patient)

### Category 2: establishing a mutual understanding

Participants raised the importance of establishing a mutual understanding in the patient–clinician encounter, by understanding the patient’s perspective and assessing the severity and criticality of the situation, as an important aspect of safety-netting advice. Their reflections suggested that, in order to establish a mutual understanding, deliberate and exploratory inquiry through both verbal and nonverbal communication is needed, and that this aspect requires further dialogue beyond what is needed for understanding the preconditions.

#### Facilitating and understanding the patient’s perspective

The participants explained that clinicians and patients may have different agendas and emphasised the importance of understanding the patient’s context and perspective. This was illustrated by one patient who said that their priority is both to ‘survive’, and also to ‘survive and thrive’. Clinicians found it useful to use established consultation techniques based on patients’ ideas, concerns and expectations.^18^ These give structure to the consultation and enable them to both capture the patient’s perspective and share their own, in an effort to establish a mutual understanding. Patients reminded the clinicians of the challenge of being in a patient–clinician consultation, which can make them forget the questions they wanted to ask and feel disempowered. This further emphasised the clinician’s responsibility to structure the consultation with open questions that help patients express their ideas and concerns.

It was emphasised that productive patient–clinician interactions are dependent on both verbal and non-verbal communication skills. The participants described how patients’ perceptions may be selective, which could lead to faulty interpretations of what the clinician intends to communicate. Notably, patients described assigning meaning to non-verbal information, such as silences.

When I think of patient safety and many other things, what actually happened…because it was survival that was the focus at that time. Those things I know now, then doctors were satisfied. But afterwards, you were supposed to go out and live your life. That was something completely different. So, surviving and thriving, not just surviving I used to say. That’s something I really want to convey. (Interview, patient)

I just find that the silence is always the worst. (Focus group, patient)

#### Assessing and communicating the severity and criticality of a situation

Emergency clinicians explained that one of the critical aspects of their job is to handle or rule out critical conditions, and sometimes solely that, which can lead to disappointment among patients whose problems remain unexplained. As one of the physicians pointed out, their work primarily deals with ‘red flags’, but the ‘orange flags’ that are less severe are more difficult to address. To make proper assessments, clinicians emphasised the importance of being able to fully understand and address a patient’s concerns. Some described looking for subtle cues and body language, and others stressed the importance of establishing a connection with the patient and determining whether there are any discrepancies in their understanding of the criticality of the situation. It was suggested that sometimes patients’ worries can be lessened by providing information and explanations, whereas in other situations patients may need to be made aware of potential risks.

I usually start by, regardless of where or in which role I am, explaining my perspective to the patient: to rule out, which means focusing on the most acute and dangerous [possibilities] first. And then, if you can move past that, then everything else is on a bonus level, if you see what I mean. How much you can actually explain in that first encounter, that is, it’s more like a bonus. But my goal, especially in the ED, no, actually also in primary care consultations (laughs), is really to think through—what’s most dangerous? What [conditions] must I not miss? (Focus group, physician)

## Discussion

This article explores the views of patients, informal caregivers and clinicians regarding how safety-netting can successfully be applied in the time-pressured contexts of primary and emergency care. Our results show that all three participant categories believe that an understanding of the preconditions for the consultation and the establishment of a mutual understanding are vital elements of successful safety-netting advice. The need for patient and clinician to form a team to accomplish successful safety-netting was evident, even though the degree of patient engagement might differ from case to case, which affects the safety-netting provided. In our interviews, both clinicians and patients reflected on the fact that, during the patient–clinician encounter and afterwards, patient and clinician form a team with the shared, complex task of following the plan created in the consultation, monitoring symptoms and acting on any deviations in how they develop. If safety-netting is viewed as a shared cognitive task, a discussion from a macrocognitive perspective might provide an enhanced understanding of the cognitive and physical work undertaken by patients and clinicians.

### Safety-netting from a macrocognitive perspective

The macrocognitive framework describes the mental activities that occur in natural settings when teams perform complex tasks,^19 20^ and has been used in analyses of teamwork among clinicians in complex and critical situations.^21^ The framework describes macrocognitive functions that include detecting problems, making sense of difficult situations, engaging in planning and replanning, making decisions and coordinating team efforts.^21^ To accomplish these activities, teams engage in macrocognitive processes such as developing mental models of situations, exploring possible future developments through mental simulations, identifying leverage points that could be turned into courses of action and maintaining common ground to coordinate activities within the team.^21 22^

Patterson *et al*^22^ describe the potential for using this macrocognitive framework as the basis for a work-system redesign aimed at reducing diagnostic delays in ambulatory care settings. However, they did not include the patient in their healthcare team. In a recent study,^23^ we report on how patients, informal caregivers and clinicians engaged in shared sensemaking and decision-making during the early phases of the pandemic. Together, this learning network of patients, clinicians and informal caregivers navigated the challenges posed during a time of great uncertainty by sharing information and exchanging strategies for replanning both self-care and healthcare situations.^23^ This need for patients and clinicians to navigate and manage uncertainty is very much present during consultations in ambulatory care settings, although on a smaller scale.

Building on the work of Patterson *et al*,^19 22^ we suggest that the aspects of safety-netting can be understood through the lens of the macrocognitive framework.

**Figure 1:**
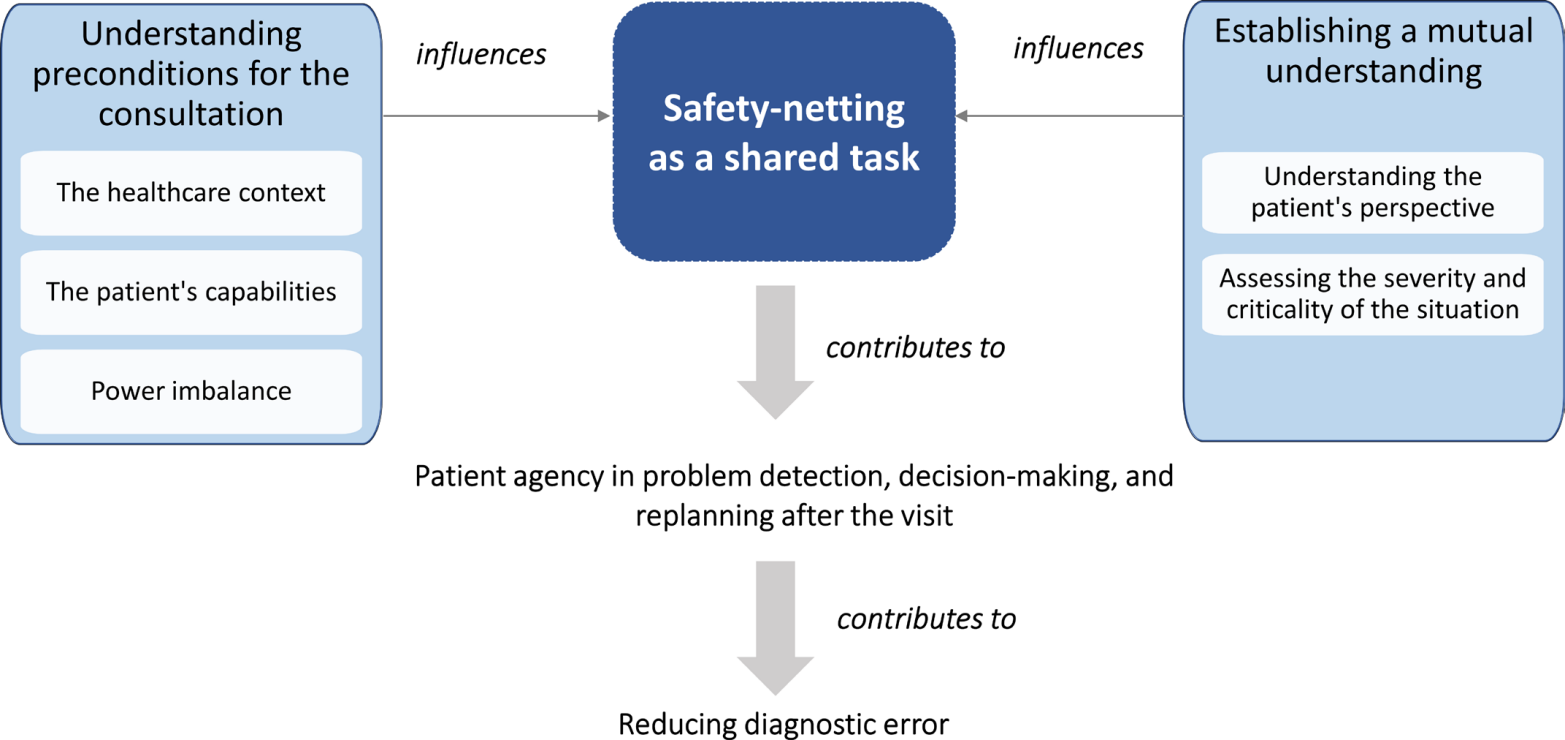
Safety-netting as a shared cognitive task in the patient–clinician team.

To further explore safety-netting as a shared cognitive task in a patient–clinician team, we integrated our findings into a model illustrating how the application of safety-netting in the patient–clinician interaction is influenced by the preconditions for the consultation and the process of establishing a mutual understanding, in an iterative process (figure 1). The elements of the figure are discussed below.

When *understanding the preconditions for the consultation*, both clinicians and patients described how they engaged in sensemaking around important aspects of their interaction with the goal of establishing a shared mental model of *the context of the healthcare setting* where the encounter takes place and *the capabilities of the patient*, while *facilitating the conversation to mitigate the power imbalance*.

Both patients and clinicians emphasised that understanding the preconditions of the consultation also involved understanding aspects of the actual patient–clinician interaction.

Previous research has explored the *content* of safety-netting,^24^ but in our study, the participants stressed that safety-netting also needs to be tailored to the surrounding clinical *context* in which it takes place. Edwards *et al*^25^ report that the nature of the care visit affects the documentation of spoken safety-netting advice in routine GP consultations. Although there was great variation in practice regarding safety-netting, GPs were more likely to document their safety-netting advice for new problems when only a single problem was discussed in a consultation, and when they gave specific rather than generic safety-netting advice. In our study, when reflecting on the concept of safety-netting, the participants raised the importance of first making sense of the context (ie, in which healthcare setting the consultation is taking place), as well as understanding the possible trajectories of care needs when advising the patient where best to seek care if a new consultation is needed. Clinicians discussed how the preconditions also differed in different healthcare settings. They described the importance of contextualising the choice of possibilities for follow-up because these differ between EDs and primary-care settings.

Both patients and clinicians also emphasised that understanding the context of the consultation involves understanding the capabilities of the patient and their network. Addressing the power imbalance between patient and clinician through conversation was seen as a vital aspect of reaching a mutual understanding of the preconditions for the consultation. The patients stated that clinicians have a great responsibility to facilitate the conversation in such a way that the patient’s perspectives and capabilities are explored. This process might be aided by communication tools, and Rake *et al* have presented promising results showing how a patient decision-making aid with the goal of fostering genuine dialogue changes the interaction in clinician–patient encounters.^26^

*Establishing a mutual understanding* by establishing a shared mental model around the patient’s understanding and capabilities, as well as the severity and criticality of the situation, is another vital aspect that must be addressed before engaging in safety-netting. Our participants emphasised the importance of exploring the patient’s awareness and understanding of the criticality of their situation, in order to empower the patient to express what was most important to them and ask questions. Patients discussed how the quality of this aspect was affected by both verbal and non-verbal communication. This shared mental model can then help to support decision-making during and after the clinical visit by highlighting possible developments and allowing the clinician and patient to develop shared strategies for problem detection and decision-making.

Verbalising the uncertainties of the diagnostic process by stating that decisions on diagnoses are not static, but rather are about making a choice among a spectrum of contingencies, has been described in previous research and was raised as an important aspect that clinician and patient both need to be aware of.^5^ The clinicians explained that this verbalisation needs varying amounts of time, depending on the capabilities of the patient and the nature of the situation. In a recent study, a programme theory was presented, suggesting mechanisms for safety-netting and providing recommendations for clinicians,^27^ although it had the disadvantage of prolonging the consultation if all the recommendations were used. The establishment of a shared mental model of a situation, as well as all the possible trajectories, may take more time, but doing so might also explain the lower occurrences of reconsultations and readmissions shown in the study by Peter *et al*.^28^ In the study, it is shown that active use of the method teach-back improved patients’ understanding of their disease and reduced readmission rates.

Our participants gave examples showing that understanding preconditions and establishing a mutual understanding are iterative processes, and that they continuously replan the strategies for safety-netting based on how the conversation develops. They also described how this replanning of safety-netting strategies is influenced by both verbal and non-verbal feedback during the clinical consultation. Replanning the strategies used for safety-netting allows the safety-netting advice to be tailored so that a mutual understanding which includes the criticality of the situation, a shared mental model of the possible trajectories of the situation, the needs and capabilities of the patient and the contextual preconditions can be established for each unique clinical encounter.

The clinicians stated that it was a challenge not knowing if the shared plan would be followed, even when they shared different strategies for checking in with patients. Both clinicians and patients noted that patients carry the greatest responsibility for cognitive tasks after the visit, and that one important aspect of safety-netting is to support patient agency in problem detection, decision-making and replanning, depending on how the situation develops. Successful and tailored safety-netting might reduce the risk of delayed or incorrect diagnoses leading to harm through extending the patient–clinician interaction and supporting the patient’s agency in problem detection, replanning and decision-making after the consultation.

#### Strengths and limitations

The strengths of this study include the contributions of the participating patients and informal caregivers, and the fact that the clinicians came from different settings. In the focus groups, the patients’ and clinicians’ discussions were enriched by views from the others in the group.

The limitations of the study include the fact that we might not have captured all aspects of the subject in the focus groups or interviews. The material reflects the views of participants belonging to a homogeneous ethnical group with a shared culture, education and language. Future research with other respondent groups would be needed to explore the transferability of our findings to other settings. The focus groups and interviews were all conducted digitally via Zoom due to both the pandemic and geographical distances, which could have affected the discussions. Although we facilitated the meetings to enable everyone to speak, there may have been an asymmetry in power between patients and clinicians in the focus groups that could have affected the results. This effect was probably mitigated by the following individual interviews.

## Conclusions

In this study, both clinicians and patients highlighted the importance of a mutual exploration of the prerequisites for the encounter before engaging in safety-netting. The establishment of a shared mental model between clinician and patient concerning the preconditions for the clinical encounter are vital factors affecting how safety-netting advice is communicated and received, and its ability to support the patient in problem detection and planning after the visit. Our results suggest that successful safety-netting can be viewed as a team-activity during which clinicians and patients collaborate in monitoring how the patient’s condition progresses after the care visit, paying particular attention to deviations from the predicted trajectory. Our findings suggest that it is important to embed safety-netting advice mindfully in clinical practice, and that it needs to be tailored to both the context of the situation at hand, and the individuals involved in the consultation. Further research is needed to assess how safety-netting can be used more often in practice and to determine optimal strategies for delivering it in a way that benefits patients as well as clinicians.

## supplementary material

10.1136/bmjopen-2023-077938 online supplemental file 1

10.1136/bmjopen-2023-077938 online supplemental file 2

10.1136/bmjopen-2023-077938 online supplemental file 3

## Data Availability

Data are available upon reasonable request.
