# Supplementary material for: Patients’ and clinicians’ views on the appropriate use of safety-netting advice in consultations—an interview study from Sweden
Source: BMJ Open. 2023 Oct 5;13(10):e077938. doi: 10.1136/bmjopen-2023-077938 (PMC10565180; doi:10.1136/bmjopen-2023-077938)
Supplement: online supplemental file 1 [file bmjopen-2023-077938-s001.pdf]

Focus Group Interview Guide for Physicians and Patients (Conducted Digitally via Zoom)

Kindly consider whether you have encountered any positive or negative instances during healthcare interactions. We will commence by allowing each participant to introduce themselves and share an experience or reflection related to the subject. Our interest lies not so much in the routine occurrences at a doctor's office, but more in what you require in situations involving uncertain diagnoses, to ensure feelings of safety, both as a medical professional and as a patient, family member, or caregiver.

For Patients: What information do you consider important to receive during a consultation with a doctor? How should information about potential serious situations be presented for easy comprehension?

For Doctors: How should safety-netting information be organized (order, content, phrasing) to provide support for you during patient encounters and after the consultation?

For Patients: What information do you believe is appropriate to include in "safety-netting"?

For Doctors: How do you ensure that the information provided is helpful and not unnecessarily concerning?

For Patients: What elements should the conversation encompass to make you feel adequately supported in handling the situation? Are there any decisions that need to be made or plans for follow-up?

For Patients: How would you prefer to receive information about when and where to seek medical care again? (e.g., verbal, written, mobile app, phone call, website, QR code...)

For Doctors: How should the safety-netting information be given to the patient?

For Doctors: How do you follow up to confirm that the decisions made during the consultation have been correctly understood?

For Patients: How should information about a the level of diagnostic uncertainty be communicated?

For Patients: What are your thoughts on a doctor expressing some degree of uncertainty about the diagnosis?

For Doctors: How do you ascertain the patient's well-being between and after visits?

For Doctors: How should the utilization of safety-netting be documented in the medical record?

For Patients: What elements do you believe should be incorporated into an online training program for doctors to enhance the use of safety-netting?

For Doctors: How should the training be designed to support you in your differential diagnostic thinking and in providing safety-netting advice?

Possible Additional Questions for Patients:

What type of information would you require regarding the anticipated course of the condition? What the likely timeline for the symptoms is?

How would you prefer to receive guidance about managing the situation when the condition doesn't progress as expected?

How should information about potential warning symptoms be conveyed?

Possible Additional Questions for Doctors:

Who, in your opinion, should receive safety-netting advice?

How do you develop an understanding of what information is most crucial to convey to patients and family members? Can you describe your preparation process?

Of the identified or described steps, which ones are absolutely essential? If time is limited, which ones would you prioritize?

What tools do you utilize for preparation? (e.g., checklists, medication lists, notes, electronic medical record systems...)

Consider your actions during the actual conversation before a patient leaves. Can you outline the steps, tasks, and components involved?

Of the steps you've identified, which ones are the most important, challenging, or difficult? In what manner?

What information do you require to carry out your tasks as effectively as possible? How do you currently acquire this information?

Do you employ any tools during patient visits?

In the concluding chat: Is there anything we haven't addressed? Please feel free to share your thoughts here.
