# Supplementary material for: Patients’ and clinicians’ views on the appropriate use of safety-netting advice in consultations—an interview study from Sweden
Source: BMJ Open. 2023 Oct 5;13(10):e077938. doi: 10.1136/bmjopen-2023-077938 (PMC10565180; doi:10.1136/bmjopen-2023-077938)
Supplement: online supplemental file 2 [file bmjopen-2023-077938-s002.pdf]

Patient Interview Guide (Conducted Digitally via Zoom)

What information is important for you to receive during a consultation with a doctor?

What does the conversation need to include for you to feel that you have the support you need to manage the situation? (Prompts: Are there any decisions that need to be made? Plans for follow-up goals/treatment?)

Do you usually express how you want things to be?

How should information about serious situations that may arise be presented so that it's easy to understand?

What questions do you need answered in order to feel safe leaving the doctor´s office?

How should this information that you want to receive be conveyed for easy understanding?

In addition to the conversation, would you like written information as well?

What are your thoughts on a doctor expressing some level of uncertainty about the diagnosis?

How should information about some level of uncertainty regarding the diagnosis be communicated?

What type of information would you need about the expected course of the condition? Likely timeline of the symptoms?

How would you like to receive information about managing the situation when the course of the condition doesn't go as expected?

Have you experienced a situation where a doctor expressed some degree of uncertainty? How did you react to it?

We're wondering how to ensure that the information is helpful without causing unnecessary worry. Do you have any thoughts on that?

When you visit with family members and it's probably nothing serious, but relative could have a serious disease, what information or guidance would you need from the primary care center in that case?

How should information about possible warning symptoms be conveyed?

How would you like to receive information about when and where you should seek medical care again?

How can we ensure that information is helpful rather than causing unnecessary concern?

If a training program for doctors about safety-netting were to be developed, what do you think should be included?
