# Supplementary material for: Patients’ and clinicians’ views on the appropriate use of safety-netting advice in consultations—an interview study from Sweden
Source: BMJ Open. 2023 Oct 5;13(10):e077938. doi: 10.1136/bmjopen-2023-077938 (PMC10565180; doi:10.1136/bmjopen-2023-077938)
Supplement: online supplemental file 3 [file bmjopen-2023-077938-s003.pdf]

Physicians Interview Guide (Conducted Digitally via Zoom)

We begin with a patient case. A patient presents with abdominal pain, and you are uncertain about the diagnosis. It's not likely to be something serious, but it could potentially be serious.

How do you form an understanding of what is most important to inform patients/family members about? Can you describe what you do to prepare?

Of the steps [tasks|components|preparations] you have just identified/described, which ones MUST you do? If you were short on time, which ones would you prioritize?

What tools do you use when preparing? (e.g., checklists, medication lists, notes, electronic medical record systems...)

Think about what you do during the actual conversation before sending a patient home. Can you describe the steps/tasks/components you go through? (To maintain a reasonable level of detail, it's good to think in terms of around 3-6 steps.)

Of the steps [steps|tasks|components|decisions] you have just identified, which ones are the most important/difficult/challenging? In what way?

What information do you need to perform your tasks as effectively as possible? How do you currently obtain that information?

Do you use any tools during the visit?

How do you follow up to ensure that the decisions made during the conversation have been understood correctly?

How do you know how the patient is doing between visits? Do you need to know? Do you want to know?

How do you know if the proposed treatment or behavior changes that you and the patient have agreed upon are actually being implemented?

How should safety-netting information be structured to support you in the patient encounter? And to support the patient after meeting with you?

How should it be delivered?

How do you ensure that information is helpful rather than causing unnecessary concern/worry?

What warning flags/alarm symptoms should patients/family members be attentive to? (Regarding each diagnosis)

In what order should the information be presented?

How should the information be phrased?

How should it be documented in the medical record?

How could education for doctors be designed to support you in using safety-netting and enhancing your differential diagnostic thinking?
