# Supplementary material for: Patients’ and clinicians’ views on the appropriate use of safety-netting advice in consultations—an interview study from Sweden
Source: BMJ Open. 2023 Oct 5;13(10):e077938. doi: 10.1136/bmjopen-2023-077938 (PMC10565180; doi:10.1136/bmjopen-2023-077938)
Supplement: Supplementary file 4 [file bmjopen-2023-077938.reviewer_comments.pdf]

PEER REVIEW HISTORY

BMJ Open publishes all reviews undertaken for accepted manuscripts. Reviewers are asked to complete a checklist review form (**http://bmjopen.bmj.com/site/about/resources/checklist.pdf**) and are provided with free text boxes to elaborate on their assessment. These free text comments are reproduced below.

ARTICLE DETAILS

**TITLE (PROVISIONAL)**	Patients' and clinicians' views on the appropriate use of safety-netting advice in consultations – an interview study from Sweden
**AUTHORS**	Fernholm, Rita; Wannheden, Carolina; Trygg Lycke, Sofia; Riggare, Sara; Pukk Harenstam, Karin

VERSION 1 – REVIEW

**REVIEWER**	Schuttner, Linnaea<br>University of Washington
**REVIEW RETURNED**	10-Aug-2023

**GENERAL COMMENTS**	Thank you for the opportunity to review your manuscript. Below are some suggestions that I hope may be of use to strengthen your paper. In general, I found the methods sound and the discussion well written.<br><br>1. Abstract.<br>- Please define safety netting immediately, in the background, for those that are not familiar with the term.<br>- Please define what digital care is, among participant physicians.<br>- Results: sentence missing a parenthesis.<br><br>2. INTRODUCTION<br>2a. Introduction – Page 3, line 32. "…therefore a greater risk of diagnostic error than in other parts of the healthcare system (1).<br>t is not possible to extrapolate that the prevalence of harm is greater in the primary care or ED setting from a descriptive study based on a database of reported patient harms. This cited study says nothing about prevalence or proportionate risk in other settings, only that more harms are reported from these settings. I would at bare minimum suggest changing that the etiology of potential higher reporting is due to higher contact / volume i.e. simply increasing the amount of exposure, but the implied adjusted risk per encounter of error is not possible from the cited studies.<br><br>2b. Introduction – Page 3, line 38-39. Please offer more detailed or clear citation to support the statement that "diagnostic error have often more severe consequences than harm due to other causes, like medication error", as it is difficult to find the supporting evidence within the full 400+ page Academy of Sciences report.<br><br>3. METHODS:<br>3a. Please define "digital care" at first use here, as well as in abstract.<br>3b. Study design is a bit ambiguous at first, I might clarify "built on each<br>other's views on the topics" a bit more concretely.

	3c. How were individuals selected for interviews from the focus group? Why weren't the three patients who were provided individual interviews also included in focus groups?<br>3d. Consider adding reflexivity section of authors<br>3e. Please describe size and composition of focus groups<br><br>4. RESULTS.<br>4a. Page 9, line 26. "Clinicians' ability to listen, meet patients with respect, and acknowledge their concerns and feelings were emphasised."<br>- Who emphasized this, patients? Or clinicians? Switch to active voice may help.<br><br>4b. I would find it helpful to look at the interactions that emerged between patients and clinicians within focus groups – because with a topic as sensitive as diagnostic error or uncertainty, it would seem inherently limiting or altering of the data collected to have both groups interacting. For example, the sentence, "Patients reminded the clinicians of the<br>challenge of being in a patient–clinician consultation, which can make them forget the<br>questions they wanted to ask and feel disempowered" makes me wonder if this dynamic was not pervasively coloring the responses throughout. The authors allude to this in the discussion Limitations ("there may have been an asymmetry in<br>power between patients and clinicians in the focus groups that could have affected the results."), but this seems to be insufficiently explored.<br><br>5. DISCUSSION.<br>5a. Discussion line 36, page 16. It might be helpful to give a few more words about the literature by Peter et al. (reference 26), rather than simply referring to the "lower occurrences of reconsultations and readmissions shown in the study by Peter et al. (26)" for audiences that are not intimately familiar with this work.<br><br>5b. I found the connection between diagnostic uncertainty, which is set up in the introduction and highlighted as an opening strength in the bullet points about the study, and safety netting, which is the main body of the results and discussion, to be rather thin. It is primarily seemingly connected in the Discussion, page 15, line 12 ("Successful and tailored safety-netting might reduce the risk of delayed or incorrect<br>Diagnoses….") only. This seems very loosely related, and I think the paper would be strengthened by narrowing in on safety netting in the introduction, rather than setting up the framing to concentrate on diagnostic error. There are many additional mechanisms that contribute to diagnostic error, and it seems distracting as a preamble.

**REVIEWER**	Hogden, Anne<br>University of New South Wales, Centre for Clinical Governance Research
**REVIEW RETURNED**	12-Aug-2023

**GENERAL COMMENTS**	Thank you to the authors for an interesting and thought-provoking qualitative study. My comments below are suggestions to improve the detail and comprehensiveness of the manuscript.

	The abstract states that the COREQ was used, but the SRQR was provided.<br><br>Abstract - Participants: White is a colour - is there another way to state that the participants came from a single ethnicity in Sweden?<br><br>The Introduction sets up the problem well. The Methods for data collection and analysis are well described and rigorous.<br><br>Study design:<br>More detail is needed in the study design section. Use the SRQR to prompt more detail; in particular about the study team, on p.4, lines 41-42 "The research group constituted of both...". What kind of clinicians and analysis experts? What was the patient's research interests (if relevant to the methodology). Please note that the SRQR page numbers don't match the reviewer copy of the manuscript - section headings are more reliable for these kinds of checklists.<br><br>Study participants:<br>The exclusion criteria for the study are not indicated for patients and caregivers. Who did you include and exclude, and why?<br><br>Strengths and Limitations:<br>One of the limitations of this study that is not discussed is that a shared mental model relies on shared communication, and to some extent a shared culture, education and language. While this study was restricted to a single ethnicity, it would be good to see mention of how these variables may influence the model in a less heterogeneous context - as future research.<br><br>The manuscript would benefit from a review for academic English. The standard of English is very good for most of the manuscript, but a little assistance to improve high-level language is needed in some parts. An example is on p.4 in lines 41-42 :"The research group constituted of both..." and line 3 on the same page "acute ill children"

VERSION 1 – AUTHOR RESPONSE

Reviewer: 1
Dr. Linnaea Schuttner, University of Washington
Comments to the Author:
Thank you for the opportunity to review your manuscript. Below are some suggestions that I hope may be of use to strengthen your paper. In general, I found the methods sound and the discussion well written.

1. Abstract.
- Please define safety netting immediately, in the background, for those that are not familiar with the term.
Thank you, now defined.

- Please define what digital care is, among participant physicians.
We have removed the reference to “digital care” (i.e., care delivered via the internet), as digital care in our study setting belongs to the primary care level.

- Results: sentence missing a parenthesis.
This has been corrected.

2. INTRODUCTION
2a. Introduction – Page 3, line 32. "…therefore a greater risk of diagnostic error than in other parts of the healthcare system (1).
t is not possible to extrapolate that the prevalence of harm is greater in the primary care or ED setting from a descriptive study based on a database of reported patient harms. This cited study says nothing about prevalence or proportionate risk in other settings, only that more harms are reported from these settings. I would at bare minimum suggest changing that the etiology of potential higher reporting is due to higher contact / volume i.e. simply increasing the amount of exposure, but the implied adjusted risk per encounter of error is not possible from the cited studies.
Thank you for pointing that out, we have clarified this sentence.

2b. Introduction – Page 3, line 38-39. Please offer more detailed or clear citation to support the statement that "diagnostic error have often more severe consequences than harm due to other causes, like medication error", as it is difficult to find the supporting evidence within the full 400+ page Academy of Sciences report.
Thank you, we change to a better reference: Diagnostic error in a national incident reporting system in the UK by Sevdalis et al.

3. METHODS:
3a. Please define "digital care" at first use here, as well as in abstract. We have removed the reference to "digital care" (i.e., care delivered via the internet), as digital care in our study setting belongs to the primary care level.

3b. Study design is a bit ambiguous at first, I might clarify "built on each
other's views on the topics" a bit more concretely. Clarified:
An exploratory research design was used, based on focus-group discussions and individual interviews with clinicians, patients, and informal caregivers. By combining these two techniques, we were able to capture both group reflections and individual insights.

3c. How were individuals selected for interviews from the focus group? Why weren't the three patients who were provided individual interviews also included in focus groups? All participants were invited to participate in an initial focus group discussion, followed by an individual interview: 17 agreed to participate in a focus group or interview, 14 participated in the focus group only, and 7 participated in an interview only. The reason for not participate in focus-group was that they had prior engagement that day so they could not make it.

3d. Consider adding reflexivity section of authors?
Authors included physicians but also non-physician and a patient researcher (part of the research group, not a participant) which helps with the perspectives in the analysis. Reflexivity was integrated in the analytic process through iterative occasions where the possible impact of subjective experiences and knowledge of the researchers on the interpretations of the findings where discussed. Added in the end of "Data analysis".

3e. Please describe size and composition of focus groups Yes, seven per group, mixed, description added to the text

4. RESULTS.
4a. Page 9, line 26. "Clinicians' ability to listen, meet patients with respect, and acknowledge their concerns and feelings were emphasised."

- Who emphasized this, patients? Or clinicians? Switch to active voice may help.
Yes, changed to: Patients underscored the significance of doctors actively listening, treating patients with respect, and acknowledging their concerns and emotions.

4b. I would find it helpful to look at the interactions that emerged between patients and clinicians within focus groups – because with a topic as sensitive as diagnostic error or uncertainty, it would seem inherently limiting or altering of the data collected to have both groups interacting. For example, the sentence, "Patients reminded the clinicians of the challenge of being in a patient–clinician consultation, which can make them forget the questions they wanted to ask and feel disempowered" makes me wonder if this dynamic was not pervasively coloring the responses throughout. The authors allude to this in the discussion Limitations ("there may have been an asymmetry in power between patients and clinicians in the focus groups that could have affected the results."), but this seems to be insufficiently explored.
Thank you, that was a reason for performing individual interviews AFTER the focus group discussions, which we have now clarified. By triangulating data collection techniques, we could mitigate the risk that participants did not feel safe to express their opinions in full.

5. DISCUSSION.
5a. Discussion line 36, page 16. It might be helpful to give a few more words about the literature by Peter et al. (reference 26), rather than simply referring to the "lower occurrences of reconsultations and readmissions shown in the study by Peter et al. (26)" for audiences that are not intimately familiar with this work.
Yes, added: In the study it is shown that active use of the method teach-back improved patients' understanding of their disease and reduced readmission rates.

5b. I found the connection between diagnostic uncertainty, which is set up in the introduction and highlighted as an opening strength in the bullet points about the study, and safety netting, which is the main body of the results and discussion, to be rather thin. It is primarily seemingly connected in the Discussion, page 15, line 12 ("Successful and tailored safety-netting might reduce the risk of delayed or incorrect
Diagnoses….") only. This seems very loosely related, and I think the paper would be strengthened by narrowing in on safety netting in the introduction, rather than setting up the framing to concentrate on diagnostic error. There are many additional mechanisms that contribute to diagnostic error, and it seems distracting as a preamble.
In the introduction we now write: A promising intervention is structured safety-netting advice as a tool for managing clinical uncertainty, with the aim of protecting patients from harm due to diagnostic error by empowering appropriate re-consultations, and thereby still receive adequate treatment in due time.
And in the discussion: Successful and tailored safety-netting might reduce the risk of delayed or incorrect diagnoses leading to harm through extending the patient–clinician interaction and supporting the patient's agency in problem detection, re-planning, and decision-making after the consultation.

Reviewer: 2
Dr. Anne Hogden, University of New South Wales
Comments to the Author:
Thank you to the authors for an interesting and thought-provoking qualitative study. My comments below are suggestions to improve the detail and comprehensiveness of the manuscript.

The abstract states that the COREQ was used, but the SRQR was provided.
Sorry for the inconvenience, now corrected.

Abstract - Participants: White is a colour - is there another way to state that the participants came from a single ethnicity in Sweden?
Ys, now the text is: The participants were an ethnically homogeneous group, originating from Western European or Australian backgrounds.

The Introduction sets up the problem well. The Methods for data collection and analysis are well described and rigorous.

Study design:
More detail is needed in the study design section. Use the SRQR to prompt more detail; in particular about the study team, on p.4, lines 41-42 "The research group constituted of both...". What kind of clinicians and analysis experts? What was the patient's research interests (if relevant to the methodology). Please note that the SRQR page numbers don't match the reviewer copy of the manuscript - section headings are more reliable for these kinds of checklists.
Thank you. We have now added: Two of the researchers were clinicians (one general practitioner and one paediatrician) and two were health informaticians, whereof one was also a patient researcher with expertise in participatory medicine. We changed to section headings in SRQR.

Study participants:
The exclusion criteria for the study are not indicated for patients and caregivers. Who did you include and exclude, and why?
Thank you, we have added:
We included 17 participants: 8 patients and informal caregivers (whereof five female) with experience of uncertain diagnoses and serious diseases, and 9 physicians (whereof seven female) with experience of working with diagnostic uncertainties in primary care or in emergency care (Table 1).

Strengths and Limitations:
One of the limitations of this study that is not discussed is that a shared mental model relies on shared communication, and to some extent a shared culture, education and language. While this study was restricted to a single ethnicity, it would be good to see mention of how these variables may influence the model in a less heterogeneous context - as future research.
Yes, now added under Strengths and limitations:
The material reflects the views of participants belonging to a homogeneous ethnical group with a shared culture, education, and language. Future research with other respondent groups would be needed to explore the transferability of our findings to other settings.

The manuscript would benefit from a review for academic English. The standard of English is very good for most of the manuscript, but a little assistance to improve high-level language is needed in some parts. An example is on p.4 in lines 41-42 :"The research group constituted of both..." and line 3 on the same page "acute ill children".
Thank you. We have improved the language.

Reviewer: 1
Competing interests of Reviewer: None

Reviewer: 2
Competing interests of Reviewer: I have no competing interests to declare.

1. Malterud K, Siersma VD, Guassora AD. Sample Size in Qualitative Interview Studies: Guided by Information Power. Qualitative health research. 2016;26(13):1753-60.

VERSION 2 – REVIEW

**REVIEWER**	Schuttner, Linnaea<br>University of Washington
**REVIEW RETURNED**	07-Sep-2023

**GENERAL COMMENTS**	Comments addressed.

**REVIEWER**	Hogden, Anne<br>University of New South Wales, Centre for Clinical Governance Research
**REVIEW RETURNED**	14-Sep-2023

**GENERAL COMMENTS**	The authors have sufficiently addressed the issues raised to make this manuscript suitable for publication. Well done on an interesting and useful study.
